# Infective Endocarditis as a Complication of Crohn’s Disease on Immunotherapy

**DOI:** 10.7759/cureus.32847

**Published:** 2022-12-22

**Authors:** Annapoorna Singh, Ain Ejaz, Preetham S Gunta, Roopesh Sai Jakulla, Daulath Singh

**Affiliations:** 1 Internal Medicine, St. Francis Hospital, University of Kansas Health System, Lawrence, USA; 2 Internal Medicine, University of Missouri–Kansas City, Kansas City, USA; 3 Internal Medicine/Hematology-Oncology, Stormont Vail Health, Topeka, USA

**Keywords:** immunocompromised status, crohn’s disease (cd), immunotherapy, aortic valve endocarditis, infective endocarditis

## Abstract

A patient with a history of Crohn’s disease on infliximab presented to the hospital with sepsis and a new heart murmur. He was found to have native aortic valve infective endocarditis from a rare species of group D *Streptococcus*in his blood. The patient was also noted to be in an acute flare of Crohn’s disease. The hospital course was complicated by florid heart failure from acute aortic insufficiency. He eventually improved after source control and appropriate antibiotic therapy. *S. pasteuranis* bacteremia and endocarditis are attributable to the patient’s immunocompromised state as a result of infliximab treatment. While *S. pasteuranis* is infrequently grown in blood cultures, it is commonly found in normal gut flora. We hypothesize that it gained access to the bloodstream through the epithelium in the terminal ileum, which was inflamed due to an acute flare of Crohn’s disease.

## Introduction

Infective endocarditis (IE) is a significant cause of mortality and morbidity in patients despite treatment [1]. Timely diagnosis and prompt medical and surgical treatment are crucial for ensuring survival and mitigating problems associated with IE. IE usually presents with fevers, chills, night sweats, fatigue, and a new cardiac murmur on physical examination; however, it can be vague at times which can pose a challenge for clinicians to diagnose. We are presenting an interesting case of IE in a patient with Crohn’s disease by a rare pathogen, *Streptococcus gallolyticus* subspecies *pasteuranis*.

## Case presentation

A 53-year-old male with a history of Crohn’s disease on infliximab presented with complaints of nausea, vomiting, decreased appetite, and generalized body aches for two to three weeks. On presentation, vital signs were significant for a temperature of 101.5°F. Initial blood workup showed leukocytosis and elevated inflammatory markers (Table 1).

**Table 1:** Laboratory values.

Laboratory tests	Results	Reference value
White blood cell count	13.8 × 10^3^/mm^3^	3,300–8,700/mm^3^
Hemoglobin	8.6 g/dL	13.8–17.2 g/dL
Serum creatinine	1.1 mg/dL	0.8–1.0 mg/dL
C-reactive protein	7.9 mg/L	<3 mg/L

**Figure 1:**
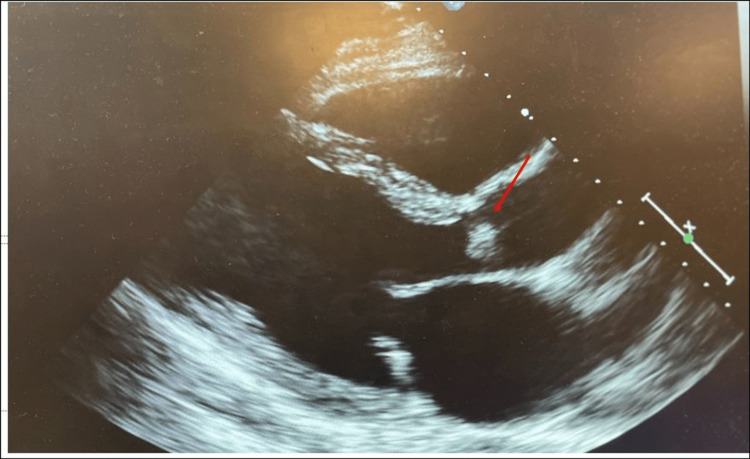
Transthoracic echocardiographic view: transverse long-axis view showing the aortic valve with a mobile echo density. Transthoracic echocardiogram depicting a mobile echo density (red arrow) attached to the aortic valve in the transverse long-axis view.

**Figure 2:**
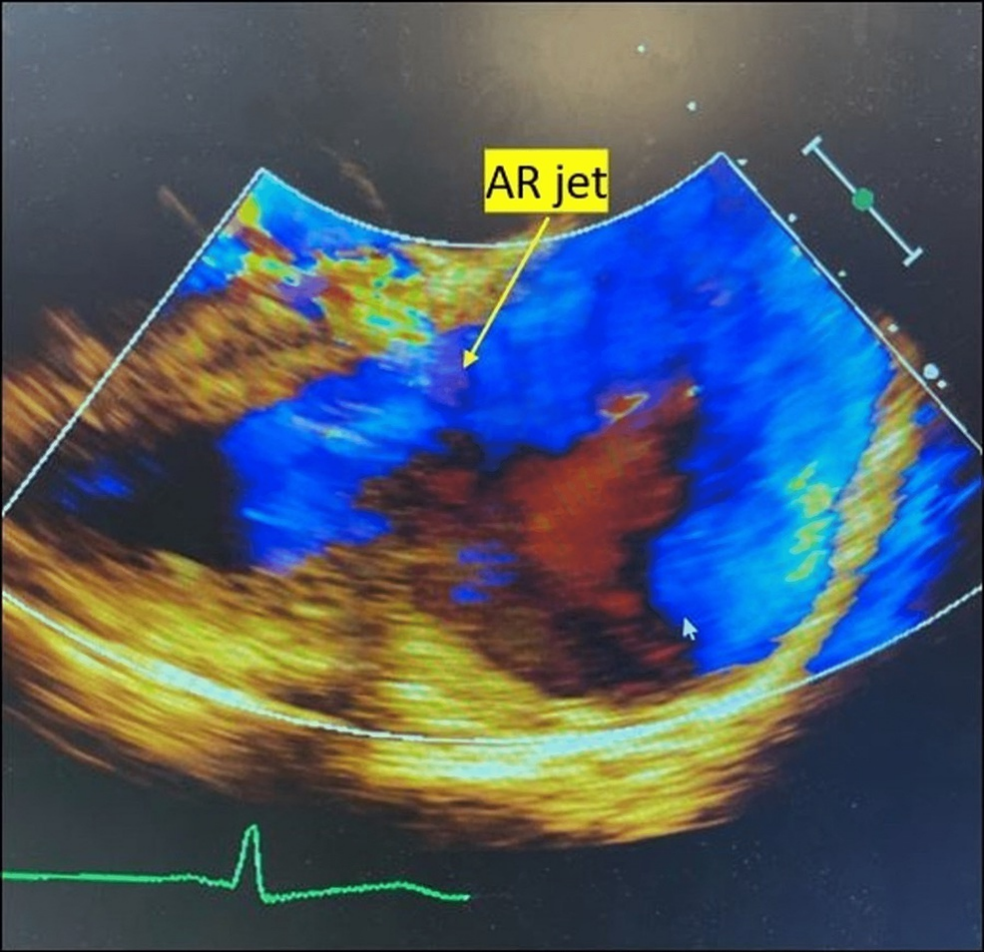
Transthoracic echocardiography showing aortic insufficiency. The echocardiogram in the parasternal long-axis view shows aortic insufficiency and aortic regurgitation (AR) jet (yellow arrow), the jet is noted to fill the left ventricular outflow tract. The flow toward the transducer is red in color, and flow away from the transducer is blue in color.

Blood cultures were drawn, and he was empirically started on levofloxacin 750 mg Q24hour for colitis concerns due to the patient’s history of Crohn’s disease. On examination, there was a murmur heard along the left sternal border. Due to the presence of murmur and fever, IE was high in the differential. Hence, a transthoracic echocardiogram was obtained, which showed an ejection fraction of 60-65% and an echo density in the right coronary cusp of the aortic valve (AV) concerning vegetation (Figure 1) and moderate-to-severe AV insufficiency (Figure 2) directed toward the anterior mitral leaflet.

**Figure 3:**
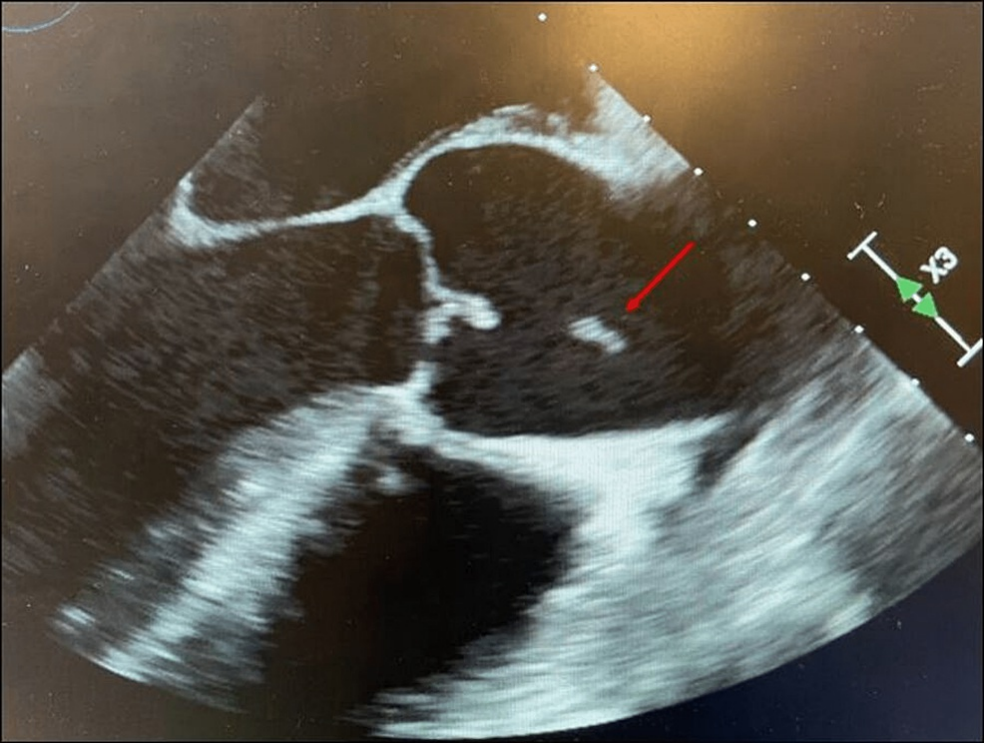
Transesophageal echocardiographic view showing the aortic valve with a mobile echo density (red arrow).

The findings were confirmed with a transesophageal echocardiogram (TEE) which also revealed 0.7 × 1.0 cm mass echo density on the right coronary cusp of the AV (Figure 3) with mild-to-moderate eccentric aortic regurgitation/aortic insufficiency (AR/AI), AI maximum velocity (MV) of 122.0 cm/second, and AI maximum peak gradient (MPG) of 50.5 mmHg.

Blood cultures grew *S. gallolyticus* subsp. *pasteuranis* which was resistant to penicillin. Levofloxacin was switched to intravenous (IV) ampicillin and IV gentamicin by the infectious disease service. The diagnosis of AV endocarditis caused by *S. gallolyticus* bacteremia with vegetation on the AV was confirmed.

**Figure 4:**
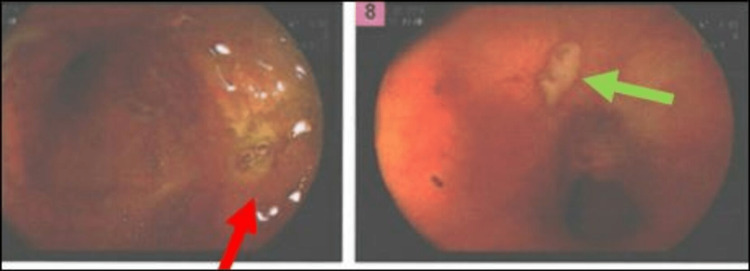
Colonoscopy images showing inflammation and ulcer in the terminal ileum. Colonoscopy depicting erythema and ulceration in the terminal ileum (red and green arrows).

The hospital course was complicated by new-onset heart failure with pedal edema and dyspnea in the setting of moderate-to-severe AR; however, surgical repair of the AV was deferred as source control was challenging in this patient because IE occurred secondary to underlying Crohn’s disease. Consequently, the patient was managed medically, and he responded well to diuretics (IV furosemide 40 mg daily) and fluid restriction. The patient underwent a colonoscopy which revealed terminal ileum inflammation and ulceration (Figure 4).

There were no polyps or discrete masses suspicious of malignancy; nevertheless, a biopsy of the terminal ileum was obtained, which showed mild active chronic inflammation of lamina propria with associated hyperplastic changes and edema. The patient was on infliximab for Crohn’s disease, which was held due to bacteremia and IE. Gastroenterology was consulted for Crohn’s management and recommended treatment with oral prednisone 50 mg daily with a gradual taper and sulfasalazine 3 g daily.

Differential diagnosis

Because the patient presented to the hospital with two weeks of generalized malaise, abdominal pain, nausea, and vomiting, and he had a history of Crohn’s disease, he was thought to be having an acute flare of Crohn’s disease. An elevated C-reactive protein was suggestive of an acute inflammatory disorder. Colitis, diverticulitis, and cholecystitis were other differential diagnoses. However, the patient had a temperature of 101.5°F and, on the physical examination, was found to have a heart murmur. IE was at the top of the differentials. Anemia is a common consequence of IE. The fever, sepsis, and bacteremia were other differentials. Once the blood cultures grew *S. pasteuranis*, all three differential diagnoses could be linked to one another, as discussed below.

Treatment

Sepsis was managed in a standard manner with fluids and antibiotics. Levofloxacin was chosen because the initial working diagnosis was an acute flare of Crohn’s disease. Subsequently, this was changed to ampicillin and gentamicin. Because the IE was complicated by aortic insufficiency and florid heart failure, surgical correction was indicated. However, surgical correction or replacement would be in vain without controlling the source of bacteremia, which was the inflamed gut. This was achieved with the help of sulfasalazine and prednisone. Meanwhile, heart failure was managed with diuretics and fluid restriction.

Outcome and follow-up

**Figure 5:**
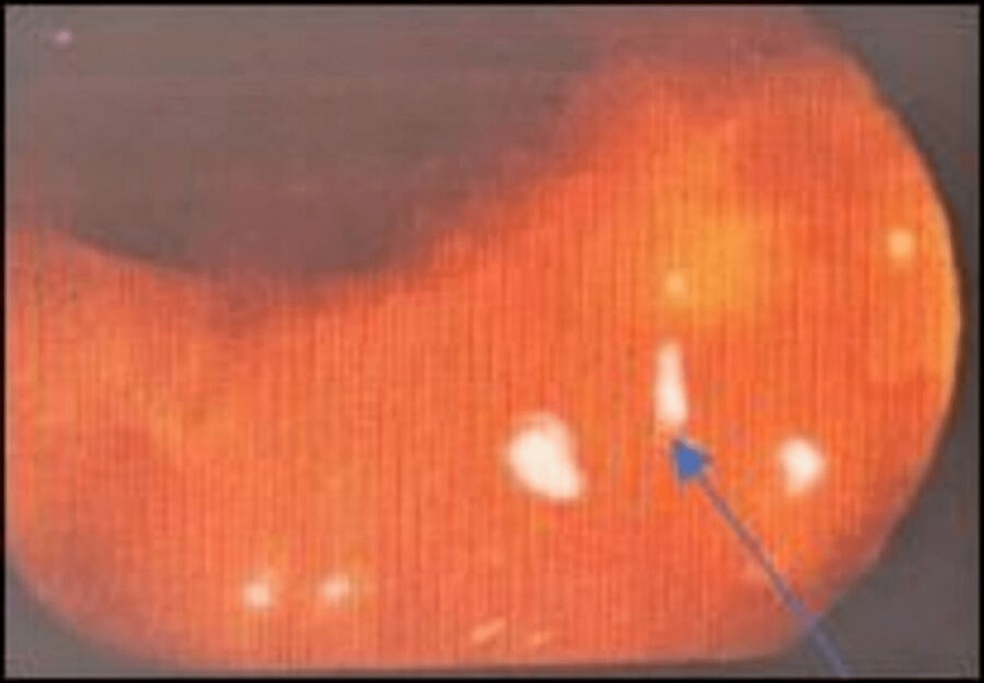
Colonoscopy images during follow-up showing the reduction in inflammation in the terminal ileum. Significant reduction in the erythema and resolution of the ulceration (blue arrow) of the terminal ileum on repeat follow-up colonoscopy.

The patient was discharged to a skilled nursing facility for completion of a six-week course of IV antibiotics. The patient underwent a repeat TEE six weeks after the initial diagnosis of IE, which revealed persistence of 0.7 × 1.0 cm echo density on the ventricular surface of the AV; however, compared to the previous TEE, the echo density appeared calcified. The AR appeared moderate but based on pressure half-time measurements; the AR was mild in nature, AI MV was 333.4 cm/second, and AI MPG was 44.5 mmHg. There was an eccentric AR jet converging on the anterior mitral valve leaflet. He also underwent a repeat colonoscopy after six weeks which showed significant improvement in the inflammation and ulceration of the ileum (Figure 5).

## Discussion

IE is an important cause of morbidity and mortality with a significant impact on healthcare. A systematic review done in 2009 suggested that despite optimal medical therapy, mortality rates from IE are around 25% [2]. Microbiologic analysis of culture-positive IE in one study showed prevalence culture positivity for *Staphylococcus aureus* in 31% of cases, viridians group streptococci in 17%, enterococci in 11%, *Streptococcus bovis* in 7%, and only 5% were other streptococci [3]. A systematic review conducted a decade later showed that the incidence of staphylococcal IE increased while that of streptococcal viridians and culture-negative IE decreased significantly in the United States [2]. Recently published studies show the emergence of other rare subspecies causes of IE including *Streptococcus acidominimus*, and *Aggregatibacter paraphrophilus* [4,5]. *S. pasteuranis* (*S. gallolyticus* subspecies *pastueranis*) is a non-enterococcal group D streptococcus commonly present as part of the gut flora [6]. It has been known to cause a variety of infectious diseases ranging from IE, neonatal sepsis, adult meningitis, peritonitis, biliary tract infections, and septicemia [7,8].

Depending upon risk factors, the microbiological profile of IE also varies [9,10]. *S. pasteuranis* and its associations with diseases in humans are largely underreported, and an increasing number of studies have been published in recent years. It has been shown to be associated with gastrointestinal malignancies, including pancreatic, gastric, hepatobiliary, and colorectal cancer with bacteremia [11,12]. As with the more commonly associated IE causative organisms, further testing and antibiotic susceptibility remain an issue with the less frequently isolated organisms. Another important aspect with regard to the underreported cases of *S. pasteuranis* is the potential drug resistance, as reported in multiple studies [13]. A retrospective study done in 2016 to assess the accuracy of the identification of *S. pasteuranis* and antibiotic resistance showed Bruker Biotyper to be the most accurate modality, 31.8% of strains were resistant to both erythromycin and clindamycin with positivity for resistance-conferring *ermB *gene [14].

An interesting aspect of the resistance pattern was based on geographic distribution [15]. A study done in China revealed higher resistance to macrolides and clindamycin [14,16,17]. This type of resistance pattern is alarming, especially in conjunction with the potential to cause serious diseases such as IE. Our patient was found to have penicillin-resistant species and his antibiotic regimen was tailored accordingly. Region-based resistance patterns will help guide the treatment for bacteremia caused by *S. pasteuranis* in the future. Another important aspect of *S. pasteuranis* IE is the screening for malignancies as it is noted to be associated with gastrointestinal malignancies, as mentioned above. Our patient underwent a colonoscopy, and terminal ileum biopsies were obtained which fortunately were negative for malignancy. As *S. pasteuranis* infections usually originate from the gastrointestinal tract, our patient’s underlying Crohn’s disease and immunocompromised status (being on infliximab) could have predisposed him to bacteremia and IE. Despite advances in medical therapy including antibiotics and early surgical intervention in patients with acute IE, mortality continues to be high due to the increasing burden of IE in healthcare and the increasing presence of comorbidities. Mortality has been noted to be higher for IE cases presenting to tertiary care facilities [18].

## Conclusions

*Streptococcus pasteuranis* (group D streptococci) are underreported causes of IE. *S. pasteuranis* usually disseminate from the gastrointestinal tract. Because *S. pasteuranis* IE has been associated with gastrointestinal tract cancers, screening for gastrointestinal malignancies is pivotal. However, other gastrointestinal diseases could also predispose to *S. pasteuranis *bacteremia. Immunosuppressive medications, such as biological agents, should be discontinued promptly in instances of bacteremia to prevent infection propagation. Specialty services should be contacted immediately for the treatment of underlying predisposing conditions (in our case, Crohn’s disease) because source control is imperative.
